# Bridging Communication Gaps: A Study on Effective Patient Communication Among Respiratory Therapy Students and Interns

**DOI:** 10.7759/cureus.60484

**Published:** 2024-05-17

**Authors:** Asail Almotery, Atheer A Bahamil, Haya S Alsehli, Rula A Alomari, Muhammad A Khan, Raju S Kumar

**Affiliations:** 1 Department of Respiratory Therapy, College of Applied Medical Sciences, King Saud bin Abdulaziz University for Health Sciences (KSAU-HS) and King Abdullah International Medical Research Center (KAIMRC), Jeddah, SAU; 2 Department of Medical Education, College of Medicine, King Saud bin Abdulaziz University for Health Sciences (KSAU-HS) and King Abdullah International Medical Research Center (KAIMRC), Jeddah, SAU; 3 Department of Basic Sciences, College of Science and Health Professions, King Saud bin Abdulaziz University for Health Sciences (KSAU-HS) and King Abdullah International Medical Research Center (KAIMRC), Jeddah, SAU

**Keywords:** respiratory therapy, interns, communication, awareness, attitudes

## Abstract

Introduction

Effective communication in healthcare plays a pivotal role, significantly impacting patient experiences and outcomes. While much of the current literature focuses on communication dynamics among physicians and nurses, a gap exists in understanding these dynamics within allied health professions such as respiratory therapy. This study explores the knowledge, attitudes, and awareness of patient communication among respiratory therapy students and interns.

Methods

This descriptive cross-sectional study investigated the knowledge, attitudes, and awareness of effective communication methods with patients among respiratory therapy students and interns in Jeddah, Saudi Arabia. Using a validated self-administered questionnaire, the study surveyed 350 individuals from three universities and associated hospitals.

Results

The analysis involved 350 participants, with females comprising 55.1%. The study found that the highest level of agreement (mean 4.6±0.62) was regarding essential knowledge related to introducing respiratory therapists to patients during communication. Female students demonstrated significant proficiency in concluding patient interviews (P=0.033), while male students excelled in comprehending communication methods with unconscious patients (P=0.010). Interns exhibited the most comprehensive understanding of patient communication skills, particularly in employing open-ended questions (P=0.009) and allowing adequate time for patients to express their concerns (P=0.020). Gender and academic progression were identified as factors influencing patient communication skills in respiratory therapy students and interns.

Conclusion

This study highlights the need for tailored communication training for respiratory therapy students and interns. It emphasizes the importance of enhancing proficiency in this vital field by addressing knowledge gaps and identifying areas for improvement.

## Introduction

Communication is a multifaceted process wherein individuals convey their emotions and experiences to others, also serving as a channel for expressing concerns about others' lives. This process involves both spoken language and nonverbal signals. Body language is a significant component, allowing people to convey messages without relying on words [1]. It encompasses a range of nonverbal elements, including facial expressions, eye contact, gestures, physical contact, shifts in posture, and body positioning [2]. Research studies have underscored the importance of nonverbal communication in the objective structured clinical examination (OSCE) administered to medical students [2]. These studies suggest that the tone of voice utilized in verbal exchanges can carry significance, enhancing interpersonal communication [3]. Albert Mehrabian, a psychologist, identifies three crucial components in face-to-face human interaction: nonverbal behavior, vocal tones, and verbal content [3]. Widely accepted among psychologists, the 7-55-33 rule outlines the distribution of communication elements, attributing 7% to words, 55% to body language, and 33% to vocal variation [3]. In healthcare environments, the body language of providers can express a spectrum of emotions, including warmth and support, as well as signs of disinterest, irritation, or boredom [4]. Healthcare providers' communication abilities encompass collecting information, offering accurate advice, delivering treatment instructions, and establishing empathetic connections with patients [5]. Research has highlighted that positive nonverbal communication between healthcare providers and patients has been associated with favorable outcomes [6]. Throughout interactions between healthcare providers and patients, a multitude of messages are conveyed through nonverbal means [1]. Studies indicate that physicians' nonverbal communication benefits patients' pain tolerance, expression, and recall [7]. Alongside verbal and nonverbal cues, empathy plays a vital role in doctor-patient communication and can enhance patient satisfaction [8]. Empathy toward patients is a critical communication skill for healthcare professionals [9]. Research shows that nonverbal communication surpasses verbal communication in conveying empathy [10]. Effective verbal communication skills are essential for therapists when interacting with unconscious patients. Researchers have also highlighted the advantageous impact of verbal communication on unconscious patients in intensive care units (ICUs) [11,12]. Research indicates that early communication between respiratory therapists and patients significantly improves patient experience. Professionals recommend that therapists explain the typical decline in lung function to aid patient comprehension [13]. Effective communication skills are crucial for therapists to build trust with patients [14]. Clear communication among respiratory therapists, physicians, and nurses is vital for optimal patient therapy outcomes. A study on health literacy training with first-year respiratory therapy (RT) students in the United States demonstrated significant benefits in understanding effective patient communication [15].

This study aims to tackle a significant research gap by examining the knowledge, attitudes, and awareness regarding effective patient communication, especially among RT students and interns. Although existing literature predominantly examines communication dynamics among physicians and nurses, there is limited understanding of similar dynamics within allied health professions like RT. This study aims to address this gap by focusing on RT students and interns, aiming to pave the way for tailored communication training programs to improve patient communication skills.

## Materials and methods

Participants

This cross-sectional study explored the knowledge, attitudes, and awareness of effective patient communication among RT students and interns in Jeddah, Saudi Arabia. Participants were recruited from three universities, King Saud bin Abdulaziz University for Health Sciences (KSAU-HS), King Abdulaziz University (KAU), and Batterjee Medical College (BMC), along with their affiliated hospitals located in Jeddah, Saudi Arabia. Three hundred and fifty participants, encompassing both male and female third- and fourth-year RT students and interns, willingly participated in the survey after being thoroughly briefed on the study's objectives. The study took place from July 2022 to July 2023.

Sampling technique

This study utilized quota sampling. Quota sampling is a research technique that involves selecting participants according to predetermined characteristics or quotas. In this method, researchers identify specific traits within the population of interest and select participants accordingly.

Sample size

To calculate the required sample size for our research, we utilized the Raosoft® software accessible at www.raosoft.com/samplesize.html. The minimum sample size needed to attain a 95% confidence level, a response distribution of 50%, and a margin of error of ±5% was determined to be 188. However, we collected data from 350 (n=350) participants. One hundred and fifty-two were from BMC, 116 were from KSAU-HS, and 82 were from KAU.

Validity

Four subject experts, working independently, evaluated the questionnaire's content validity. Additionally, medical education specialists assessed its face validity.

Reliability

After distributing the study questionnaire to 36 individuals who were not part of the study group, we assessed reliability and found a Cronbach's alpha coefficient of α=0.91 upon analyzing their responses.

Questionnaire

To counter acquiescence bias, we incorporated both positively and negatively worded items in the questionnaire (see Appendices). Negative items were subjected to reverse scoring to prompt respondents to carefully consider the questions rather than consistently selecting the same response option. After data collection, the scores for the reverse-scored items were adjusted to align with the scoring of the other questionnaire items. 

To ensure participants' comprehension, a brief paragraph outlined the aims and objectives of the study before introducing the questionnaire.

The questionnaire had four sections. Section 1 contained questions related to demographic characteristics. Section 2 had questions that evaluated the attitudes of students and interns toward effective patient communication. Section 3 included questions that assessed the knowledge of students and interns about effective patient communication. Section 4 contained queries to evaluate the awareness of students and interns regarding effective patient communication. Except for Section 1, which covers the demographic features of the participants, all questions in other sections of the questionnaire were assessed using a five-point Likert scale, spanning from strongly disagree to strongly agree. 

Ethical clearance

The Institutional Review Board (IRB) of King Abdullah International Medical Research Center (KAIMRC) approved the self-administered validated questionnaire under the reference number SP22J/130/08. All participants provided informed consent for the study, and we ensured the privacy of their responses.

Data collection

The questionnaire was distributed through a Google survey form and shared online via WhatsApp and emails.

Statistical analysis

Data collection was conducted through Google Forms (Google, United States), with responses recorded on an Excel sheet (Microsoft Corporation, United States). Analysis of the data was performed using IBM SPSS Statistics for Windows, Version 20.0 (Released 2011; IBM Corp., Armonk, New York, United States). Descriptive analysis was provided by calculating the frequency and percentage of categorical variables, including gender, city of residence, and questions related to knowledge, attitude, and awareness. The chi-squared test/Fisher exact test was employed to compare two categorical variables, as appropriate. Furthermore, associations between the knowledge, attitudes, and awareness of students and interns with the responses were examined using ANOVA and an independent t-test. Results with a p-value of less than 0.05 were considered significant.

## Results

Attitudes

In Table 1, the lowest mean score (1.9±0.93) was for the statement "I do not show concern and care about my patient's health." Other statements such as "During my communication, I do not express empathy toward my patients," "I don't use closed-ended questions," and "I do not allow the patient to take control of the conversation" had means of 2.3±0.99, 2.5±1.14, and 2.5±1.00, respectively, indicating participants' solid understanding of these questions.

**Table 1:** Mean scores of participants' comprehension of the attitudes section of the questionnaire (n=350) SD: standard deviation

Statements concerning participants' attitudes	Mean	SD
I introduce myself to the patient with a gentle smile	4.3	0.70
I do not maintain eye contact when I communicate with patients	2.2	1.01
During communication with my patients, I actively listen to my patient's concerns	4.3	0.72
I do not show my concern and care about my patient's health	1.9	0.93
I try to use open-ended questions, for example, "Tell me how you feel today?"	4.1	0.81
I don't use closed-ended questions, for example, "Is your pain restricting your movement?"	2.5	1.14
I always try to give my patients enough time for them to speak and explain their problems	4.4	0.65
During my communication, I take into consideration the patient's customs and traditions	4.0	0.98
During my communication, I do not express empathy (caring) toward my patients	2.3	0.99
I try to comfort the patient by using examples from the Holy Quran	3.4	1.05
I do not allow the patient to take control of the conversation	2.5	1.00
I encourage my patients to ask any questions at the end of the communication	4.2	0.83

Knowledge

Introducing respiratory therapists to patients during the initial encounter garnered the highest average rating of 4.6±0.62 for knowledge in Table 2. Conversely, four negative questions, including maintaining eye contact during patient communication, allowing sufficient time for patients to express concerns, sustaining patient attention, and addressing cultural differences, received the lowest mean scores of 2.2±1.08, 2.2±1.24, 2.2±1.12, and 2.4±1.18, respectively. This suggests a robust comprehension by the participants regarding these communication elements.

**Table 2:** Mean scores of participants' comprehension of the knowledge section of the questionnaire (n=350) SD: standard deviation

Statements concerning participants' knowledge	Mean	SD
During the first visit, the respiratory therapist should introduce himself/herself to the patient	4.6	0.62
While communicating with patients, it is not very essential to maintain good eye contact	2.2	1.08
Good facial expressions (like gentle smiling) of the respiratory therapist positively influence the communication process with patients	4.2	0.75
The voice tone and vocal variety used by the respiratory therapist while communicating with the patient ensure patient cooperation during treatment	4.0	0.91
Asking appropriate questions to patients related to the clinical condition of the patient during the history-taking enhances effective treatment outcomes	4.3	0.66
Giving the patient sufficient time to explain their concerns is not important during patient communication	2.2	1.24
Using hand gestures is essential when you communicate with patients	3.4	1.07
Demonstrating empathy (caring) to patients is an essential part of effective communication	4.1	0.85
Maintaining the patient's attention throughout patient communication is not essential	2.2	1.12
Cultural differences between patients and respiratory therapists do not affect communication quality	2.4	1.18
When closing a patient's interview, it is crucial to ask the patient to summarize what he/she understood	4.2	0.83

Awareness

In Table 3, the lowest mean scores were for negative questions regarding the importance of closing patient interviews, positive body language, and verbal communication with unconscious patients in the ICU, with means of 2.3±1.09, 2.3±1.20, and 2.5±1.16, respectively. This suggests participants strongly understood these aspects in the awareness section.

**Table 3:** Mean scores of participants' comprehension of the awareness section of the questionnaire (n=350) SD: standard deviation; ICU: intensive care unit

Statements concerning participants' awareness	Mean	SD
Introducing yourself to the patient is an essential interaction to build a good therapeutic practitioner-patient relationship	4.5	0.69
Poor eye contact with the patients during communication results in negative patient satisfaction	4.1	0.86
When you are performing a procedure on an unconscious patient in an ICU, it is not essential to verbally communicate	2.5	1.16
Active listening to the patient's concerns conveys respect and builds the trust of the patient toward the therapist	4.3	0.68
Patients would not be motivated to communicate if the respiratory therapist did not give them enough time to express their problems	4.1	0.79
As a respiratory therapist, I am aware of positive hand gestures	3.4	1.11
During the conversation, a respiratory therapist should actively allow patients to take control of the interview	3.3	1.08
While the respiratory therapist communicates using the same patient's dialect, it improves the communication process	3.8	1.03
Using the appropriate skills to close the interview with patients is not crucial for patient communication	2.3	1.09
Respiratory therapists should use the patient-centered approach in their communication with the patient	4.2	0.74
Leaning away and crossing their arms while communicating with patients is considered positive body language for respiratory therapists	2.3	1.20

Although there were no significant variations in attitudes between male and female participants when examining gender differences, notable distinctions were noted in the knowledge and awareness sections of the questionnaire. Female students demonstrated a better understanding of closing patient interviews (P=0.033) in one statement within the knowledge section (Table 4).

**Table 4:** Significant findings from the gender comparison from the knowledge section of the questionnaire (n=350). An independent t-test was employed ⃰P˂0.05 is considered statistically significant SD: standard deviation; CI: confidence interval

Statement related to participants' knowledge	Gender	n	Mean	SD	95% CI		P-value
When closing a patient's interview, it is crucial to ask the patient to summarize what he/she understood	Male	157	4.1	0.85	(-0.37	0.02)	0.033
	Female	193	4.3	0.81			

Two statements in the awareness section (Table 5) revealed significant differences: male participants exhibited heightened awareness of the significance of verbal communication with unconscious patients (P=0.010), while females were more inclined to agree that patients were allotted sufficient time to express their concerns (P=0.035).

**Table 5:** Significant findings from the gender comparison from the awareness section of the questionnaire (n=350). An independent t-test was employed ⃰P˂0.05 is considered statistically significant SD: standard deviation; CI: confidence interval; ICU: intensive care unit

Statement related to participants' awareness	Gender	n	Mean	SD	95% CI		P-value
When you are performing a procedure on an unconscious patient in the ICU, it is not essential to communicate verbally	Male	157	2.64	1.133	(0.077	0.564)	0.010
	Female	193	2.32	1.168			
Patients would not be motivated to communicate if the respiratory therapist did not give them enough time to express their problems	Male	157	4.01	0.824	(-0.345	-0.012)	0.035
	Female	193	4.19	0.757			

Table 6 illustrates significant findings from the comparison of knowledge, awareness, and attitudes among third-year and fourth-year RT students and interns. Interns showed a better understanding of patient communication skills, including eye contact (P=0.006), hand gestures (P=0.001), and empathy (P=0.011). They also had higher awareness of positive communication behaviors like active listening (P=0.025) and appropriate gestures (P=0.002). Interns generally had more knowledge than fourth-year students, particularly in giving patients time to express concerns (P=0.016) and understanding vocal variety and tone (P=0.001). Third-year students understood the importance of self-introduction better (P=0.011). Interns were more aware of aspects such as active listening, positive gestures, and avoiding crossed arms (P=0.01).

**Table 6:** Significant findings from the comparison of knowledge, awareness, and attitudes among third-year, fourth-year, and intern respiratory therapy students (n=350). ANOVA was employed ⃰P˂0.05 is considered statistically significant SD: standard deviation

Statements from the questionnaire			Academic year	Mean	SD	P-value
Knowledge	While communicating with patients, it is not very essential to maintain good eye contact	Third year		2.2	1.04	0.006
		Fourth year		2.4	1.18	
		Internship year		1.9	0.96	
	The respiratory therapist's tone of voice and vocal variety while communicating with the patient ensure patient cooperation during treatment	Third year		4.0	0.87	0.001
		Fourth year		3.9	1.03	
		Internship year		4.3	0.74	
	Giving patients sufficient time to explain their concerns is not important during patient communication	Third year		2.2	1.19	0.020
		Fourth year		2.4	1.27	
		Internship year		1.9	1.21	
	Using hand gestures is essential when you communicate with patients	Third year		3.3	1.14	0.001
		Fourth year		3.3	1.08	
		Internship year		3.7	0.83	
	Demonstrating empathy (caring) to patients is essential to effective communication	Third year		4.1	0.78	0.011
		Fourth year		3.9	0.99	
		Internship year		4.2	0.74	
Awareness	Introducing yourself to the patient is essential to building a good therapeutic practitioner-patient relationship	Third year		4.7	0.53	0.011
		Fourth year		4.5	0.76	
		Internship year		4.4	0.78	
	Active listening to the patient's concerns conveys respect and builds the patient's trust toward the therapist	Third year		4.2	0.75	0.025
		Fourth year		4.3	0.60	
		Internship year		4.4	0.63	
	As a respiratory therapist, I am aware of positive hand gestures	Third year		3.3	1.13	0.002
		Fourth year		3.4	1.14	
		Internship year		3.8	0.96	
	While the respiratory therapist communicates using the same patient's dialect, it improves the communication process	Third year		3.7	1.10	0.002
		Fourth year		3.8	1.01	
		Internship year		4.1	0.87	
	When the respiratory therapist leans away with crossed arms during communication with the patient, it is considered a sign of positive body language	Third year		2.3	1.19	0.013
		Fourth year		2.6	1.20	
		Internship year		2.1	1.16	
Attitudes	I do not maintain eye contact when I communicate with patients	Third year		2.1	0.88	0.008
		Fourth year		2.5	1.10	
		Internship year		2.1	1.03	
	I try to use open-ended questions, for example, "Tell me how you feel today?"	Third year		4.1	0.78	0.009
		Fourth year		3.9	0.85	
		Internship year		4.2	0.79	
	I don't use closed-ended questions, for example, "Is your pain restricting your movement?"	Third year		2.4	1.07	0.003
		Fourth year		2.8	1.18	
		Internship year		2.4	1.15	
	I always give my patients enough time to speak and explain their problems	Third year		4.4	0.62	0.016
		Fourth year		4.3	0.65	
		Internship year		4.5	0.67	
	During my communication, I do not express empathy (caring) toward my patients	Third year		2.4	1.08	0.028
		Fourth year		2.4	0.97	
		Internship year		2.1	0.83	

## Discussion

The study covered various aspects of communication skills among RT students and interns. While participants showed strong knowledge of introducing therapists to patients, awareness of eye contact and patient attention was lower. Attitudes toward patient care emphasized concern and empathy. Participants understood communication, including closing patient interviews and positive body language. Gender differences were noted, with females excelling in understanding patient interviews and males showing a stronger awareness of verbal communication with unconscious patients. Interns displayed superior understanding and awareness of patient communication skills compared to fourth-year students, focusing on eye contact, hand gestures, and empathy.

According to Lee et al., nonverbal communication techniques can facilitate active or participatory listening to engage the patient in shared decision-making while helping the healthcare provider convey compassion and empathy [16]. Studies have shown that maintaining eye contact with patients improves satisfaction [16]. This supports our research finding that participants had good knowledge, attitudes, and awareness of the significance of eye contact in practitioner-patient interaction. Lawrence et al. found that patients' engagement and communication with healthcare professionals could be improved through consciousness-raising interventions [17]. When dealing with coma patients, communication can be challenging due to the patient's lack of responsiveness. Healthcare providers often have limited knowledge of the needs of unconscious patients, which can make it difficult to provide proper care. This raises questions about the necessity of communication in such cases [17]. It is believed that communicating with unconscious patients is crucial. Lawrence et al. conducted a study in which they interviewed patients who had recovered from a coma to explore their auditory experiences while unconscious. Most patients had no recollection of the coma, while eight had some partial subsequent memory, and 17 remembered hearing auditory stimuli during their coma [17]. The findings of these researchers validate our hypothesis that verbal communication with unconscious patients in the ICU is sufficient. Our study participants demonstrated a notable level of awareness regarding this inquiry. The research conducted by Mohd Salim and colleagues aligns with our findings regarding using open-ended questions. In their study, participants emphasized the significance of open-ended questions during patient communication to gather comprehensive information from patients and their families during consultations [18].

Receiving spiritual care can help family members and seriously ill patients manage their anxiety, stress, and despair. The healthcare personnel are not adequately prepared, and health management is not allocating all the necessary resources [19]. According to research carried out in Greece, religiosity is correlated with enhanced health outcomes, suggesting its efficacy as a viable coping mechanism for individuals facing health-related challenges [20]. Research conducted in Algeria during the COVID-19 pandemic highlighted the Muslim community's reliance on religion, beliefs, and spirituality for coping [21]. Saudi Arabia's status as an Islamic country with distinct cultural, traditional, and religious values presents a unique context. These results suggest that incorporating Islamic cultural practices into patient communication could potentially yield positive effects on compliance and health outcomes when treating Muslim patients in Saudi Arabia. Nevertheless, participants in the study displayed a low attitude toward using religious approaches to comfort patients.

According to a prior investigation, nurses typically assume a leading role during patient interactions [22]. Results indicated that participants in our study exhibited a diminished inclination toward permitting patients to take the lead in discussions. The results of our study indicate that the participants recognized the significance of respiratory therapists introducing themselves to patients.

Interns exhibited a notable grasp of the importance of positive hand gestures in patient communication compared to third- and fourth-year RT students. Research has demonstrated that gestures play a crucial role in communication and can convey a broad range of meanings and ideas beyond what language alone can express [23]. We believe using hand gestures can improve communication between respiratory therapists and their patients, enhancing overall understanding.

According to a study, Saudi nurses were perceived by patients to possess outstanding interpersonal skills [24]. Nonetheless, non-Saudi nurses were identified as more adept in certain aspects, particularly nonverbal communication, such as smiling. The patients in the study emphasized the significance of effective communication in alleviating stress related to their illnesses. They recommended that Saudi nurses undergo training to smile at patients [24].

One of our research inquiries focused on the importance of cultural variances in patient communication. Participants displayed a strong understanding of this aspect. This study underscores the necessity for additional investigation into this matter in future studies to enhance this aspect of the Saudi healthcare system, ultimately resulting in more effective patient communication.

Limitations and recommendations

It is important to note that the study has limitations when viewed globally. First, the cross-sectional design does not allow for the determination of causality between variables. Second, the study relied on self-reported questionnaire responses, which increases the possibility of recall bias, a common issue in survey-based research worldwide. Lastly, the study's geographical scope was limited to only three universities in Jeddah, Saudi Arabia, which may impact the generalizability of the findings. Future studies should consider longitudinal designs and more extensive sampling to enhance the generalizability of results and obtain a comprehensive understanding of viewpoints across different regions within Saudi Arabia. Furthermore, investigating cultural and communication disparities among Middle Eastern countries and variances among various healthcare professions would be valuable. Including other countries in future research endeavors would provide a broader perspective and facilitate comparisons across different cultural and healthcare contexts.

## Conclusions

This study found that gender and academic progression affect patient communication skills in RT education. Female students excel in concluding patient interviews, while male students perform better in dealing with unconscious patients. Interns have the most positive attitudes toward patient communication, emphasizing the need to use open-ended questions and prioritize patient expression. The study highlights strengths and areas for development in RT students' and interns' patient communication competencies, suggesting potential areas for curriculum enhancement and targeted training interventions.

## Appendices

Questionnaire

Section 1: Demographic Characteristics

1. Gender:

2. City of residence

3. Current academic year:

4. The hospital you are associated with:

Section 2: Students' Attitudes on Effective Patient Communication

1. I introduce myself to the patient with a gentle smile.

2. I do not maintain eye contact when I communicate with patients.

3. During communication with my patients, I actively listen to my patient's concerns.

4. I do not show my concern and care about my patient's health.

5. I try to use open-ended questions, for example, "Tell me how you feel today?".

6. I don't use closed-ended questions, for example, "Is your pain restricting your movement?".

7. I always try to give my patients enough time for them to speak and explain their problems.

8. During my communication, I take into consideration the patient's customs and traditions.

9. During my communication, I do not express empathy (caring) toward my patients.

10. I try to comfort the patient by using examples from the Holy Quran.

11. I do not allow the patient to take control of the conversation.

12. I encourage my patients to ask any questions at the end of the communication.

Section 3: Knowledge of Students and Interns About Effective Patient Communication

1. During the first visit, the respiratory therapist should introduce himself/herself to the patient.

2. While communicating with patients, it is not very essential to maintain good eye contact.

3. Good facial expressions (like gentle smiling) of the respiratory therapist positively influence the communication process with patients.

4. The voice tone and vocal variety used by the respiratory therapist while communicating with the patient ensure patient cooperation during treatment.

5. Asking appropriate questions to patients related to the clinical condition of the patient during the history-taking enhances effective treatment outcomes.

6. Giving the patient sufficient time to explain their concerns is not important during patient communication.

7. Using hand gestures is essential when you communicate with patients.

8. Demonstrating empathy (caring) for patients is an essential part of effective communication.

9. Maintaining the patient's attention throughout patient communication is not essential.

10. Cultural differences between patients and respiratory therapists do not affect communication quality.

11. When closing a patient's interview, it is crucial to ask the patient to summarize what he/she understood.

Section 4: Awareness of Students and Interns Regarding Effective Patient Communication

1. Introducing yourself to the patient is an essential interaction to build a good therapeutic practitioner-patient relationship.

2. Poor eye contact with the patients during communication results in negative patient satisfaction.

3. When you are performing a procedure on an unconscious patient in an intensive care unit (ICU), it is not essential to verbally communicate.

4. Active listening to the patient's concerns conveys respect and builds trust of the patient toward the therapist.

5. Patients wouldn't be motivated to communicate if the respiratory therapist didn't give them enough time to express their problems.

6. As a respiratory therapist, I am aware of positive hand gestures.

7. During the conversation, a respiratory therapist should actively allow patients to take control of the interview.

8. While the respiratory therapist communicates using the same patient's dialect, it improves the communication process.

9. Using the appropriate skills to close the interview with patients is not crucial for patient communication.

10. Respiratory therapists should use the patient-centered approach in their communication with the patient.

11. Leaning away and crossing their arms while communicating with patients is considered positive body language for respiratory therapists.

## References

[1] Wanko Keutchafo EL, Kerr J, Jarvis MA (2020). Evidence of nonverbal communication between nurses and older adults: a scoping review. BMC Nurs.

[2] Park SG, Park KH (2018). Correlation between nonverbal communication and objective structured clinical examination score in medical students. Korean J Med Educ.

[3] Mehrabian A, Ferris SR (1967). Inference of attitudes from nonverbal communication in two channels. J Consult Psychol.

[4] Berman AC, Chutka DS (2016). Assessing effective physician-patient communication skills: "are you listening to me, doc?". Korean J Med Educ.

[5] Ranjan P, Kumari A, Chakrawarty A (2015). How can doctors improve their communication skills?. J Clin Diagn Res.

[6] Ambady N, Koo J, Rosenthal R, Winograd CH (2002). Physical therapists' nonverbal communication predicts geriatric patients' health outcomes. Psychol Aging.

[7] Ruben MA, Blanch-Hartigan D, Hall JA (2017). Nonverbal communication as a pain reliever: the impact of physician supportive nonverbal behavior on experimentally induced pain. Health Commun.

[8] Vogel D, Meyer M, Harendza S (2018). Verbal and non-verbal communication skills including empathy during history taking of undergraduate medical students. BMC Med Educ.

[9] Moudatsou M, Stavropoulou A, Philalithis A, Koukouli S (2020). The role of empathy in health and social care professionals. Healthcare (Basel).

[10] Brugel S, Postma-Nilsenová M, Tates K (2015). The link between perception of clinical empathy and nonverbal behavior: the effect of a doctor's gaze and body orientation. Patient Educ Couns.

[11] Jesus LM, Simões JF, Voegeli D (2013). Verbal communication with unconscious patients. Acta Paul Enferm.

[12] Alasad J, Ahmad M (2005). Communication with critically ill patients. J Adv Nurs.

[13] Ngwenya N, Crang C, Farquhar M (2021). Communicating uncertainty: contrasting the communication experiences of patients with advanced COPD and incurable lung cancer. Fam Pract.

[14] Duffy FD, Gordon GH, Whelan G (2004). Assessing competence in communication and interpersonal skills: the Kalamazoo II report. Acad Med.

[15] Ogrodnick MM, Feinberg I, Tighe E, Czarnonycz CC, Zimmerman RD (2020). Health-literacy training for first-year respiratory therapy students: a mixed-methods pilot study. Respir Care.

[16] Lee T, Lin EC, Lin HC (2022). Communication skills utilized by physicians in the pediatric outpatient setting. BMC Health Serv Res.

[17] Lawrence MM, Ramirez RP, Bauer PJ (2023). Communicating with unconscious patients: an overview. Dimens Crit Care Nurs.

[18] Mohd Salim NA, Roslan NS, Hod R, Zakaria SF, Adam SK (2023). Exploring critical components of physician-patient communication: a qualitative study of lay and professional perspectives. Healthcare (Basel).

[19] Badanta B, Rivilla-García E, Lucchetti G, de Diego-Cordero R (2022). The influence of spirituality and religion on critical care nursing: an integrative review. Nurs Crit Care.

[20] Plakas S, Boudioni M, Fouka G, Taket A (2011). The role of religiosity as a coping resource for relatives of critically ill patients in Greece. Contemp Nurse.

[21] Achour M, Souici D, Bensaid B, Binti Ahmad Zaki N, Alnahari AA (2021). Coping with anxiety during the COVID-19 pandemic: a case study of academics in the Muslim world. J Relig Health.

[22] Huang Q, Pun J, Huang S (2022). Using a mixed-methods needs analysis to ensure the sustainability and success of English for nursing communication courses: improving nurse-patient engagement practices in globalized health care. Sustainability.

[23] Kang S, Tversky B (2016). From hands to minds: gestures promote understanding. Cogn Res Princ Implic.

[24] Alshammari M, Duff J, Guilhermino M (2022). Adult patient communication experiences with nurses in cancer care settings: a qualitative study. BMC Nurs.

